# Semi-quantitative FDG parameters predict survival in multiple myeloma patients without autologous stem cell transplantation

**DOI:** 10.1186/s40644-023-00625-z

**Published:** 2023-10-27

**Authors:** Hyunjong Lee, Seung Hyup Hyun, Young Seok Cho, Seung Hwan Moon, Joon Young Choi, Kihyun Kim, Kyung-Han Lee

**Affiliations:** 1 Department of Nuclear Medicine, Samsung Medical Center, Sungkyunkwan University School of Medicine, 81 Irwon-ro, Gangnam-gu, Seoul, 06351 Republic of Korea; 2 Division of Hematology/Oncology, Department of Medicine, Samsung Medical Center, Sungkyunkwan University School of Medicine, 81 Irwon-ro, Gangnam-gu, Seoul, 06351 Republic of Korea

**Keywords:** Multiple Myeloma, Autologous stem cell transplantation, FDG PET/CT, Revised international staging system, Prognosis

## Abstract

**Background:**

F-18 fluorodeoxyglucose positron emission tomography/computed tomography (FDG PET/CT) is useful in multiple myeloma (MM) for initial workup and treatment response evaluation. Herein, we evaluated the prognostic value of semi-quantitative FDG parameters for predicting the overall survival (OS) of MM patients with or without autologous stem cell transplantation (ASCT).

**Methods:**

Study subjects comprised 227 MM patients who underwent baseline FDG PET/CT. Therein, 123 underwent ASCT while 104 did not. Volumes of interest (VOIs) of bones were drawn on CT images using a threshold of 150 Hounsfield units. FDG parameters of maximum standardized uptake value (SUVmax), mean SUV (SUVmean), metabolic tumor volume (MTV), total lesion glycolysis (TLG), and number of focal lesions (FLs) were measured. Kaplan-Meier survival analysis with log-rank tests and Cox proportional hazards regression analyses were performed for overall survival (OS).

**Results:**

In the ASCT cohort, R-ISS stage, MTV, and TLG were associated with survival. In the non-ASCT cohort, however, R-ISS stage was not associated with patient outcomes. In contrast, high SUVmax, SUVmean, MTV, TLG, and FL could predict worse OS (hazard ratio [HR] = 2.569, 2.649, 2.506, 2.839, and 1.988, respectively). Importantly, combining FDG parameters with R-ISS stage provided a new risk classification system that discriminated worse OS in the non-ASCT cohort significantly better than did R-ISS stage alone.

**Conclusions:**

In the non-ASCT cohort, semi-quantitative FDG parameters were significant predictors of worse OS. Furthermore, combining FDG parameters with R-ISS stage may provide a new risk staging system that can better stratify the survival of MM patients without ASCT.

**Supplementary Information:**

The online version contains supplementary material available at 10.1186/s40644-023-00625-z.

## Background

Multiple myeloma (MM) is the second most prevalent hematologic malignancy and is characterized by the proliferation of malignant plasma cells [1, 2]. Diagnosis requires the identification of ≥ 10% clonal bone marrow plasma cells or biopsy-proven plasmacytoma, in addition to disease-defining events such as anemia and biomarkers of malignancy [3]. Patients with MM are initially treated with chemotherapeutic agents and proteasome inhibitors [4]. Despite advances in management, however, the prognosis of patients at higher risk remains poor [5].

The International Staging System (ISS) was developed in 2005 to classify patient risk based on serum levels of β2 microglobulin and albumin [6]. The Revised International Staging System (R-ISS) later included cytogenetic and serum lactate dehydrogenase (LDH) data for improved stratification [5]. However, it primarily relies on clinical and laboratory parameters but does not incorporate detailed genetic and molecular information. In addition, it does not consider the presence of extramedullary disease. Furthermore, it does not account for changes in disease progression or response to treatment, nor does it fully reflect the impact of newer treatments on survival. This obviously includes the prognostic impact of ASCT. There is thus a need for additional tools to better classify survival risk in MM patients.

F-18 fluorodeoxyglucose positron emission tomography/computed tomography (FDG PET/CT) provides high-contrast imaging of hypermetabolic bone lesions and extramedullary disease (EMD). It not only allows surveillance of the entire skeleton but also detects extramedullary lesions and provides important information regarding treatment response and prognosis. Clinical guidelines recommend FDG PET/CT for initial assessment and residual disease evaluation of MM [4]. In addition to its diagnostic utility, FDG PET/CT also provides valuable prognostic information. In MM, maximum standardized uptake value (SUVmax), metabolic tumor volume (MTV), and number of focal lesions (FLs) have been shown to offer useful information for predicting patient outcomes [7–9].

However, the relative values of semi-quantitative FDG parameters for MM risk stratification have not been compared. Moreover, given the significant extension of survival by incorporation of autologous stem cell transplantation (ASCT), it is important to clarify the added value of FDG parameters beyond those provided by R-ISS in patients categorized into ASCT-treated and nontreated cohorts.

In this study, we thus evaluated the prognostic value of FDG parameters derived from PET/CT images of MM patients. The ability of FDG parameters to stratify survival risk was compared to that of R-ISS stage in ASCT-treated and non-treated ASCT groups. Furthermore, we investigated whether combining FDG parameters with R-ISS stage could improve risk stratification in patients without ASCT.

## Methods

### Subjects and clinical data

**Fig. 1:**
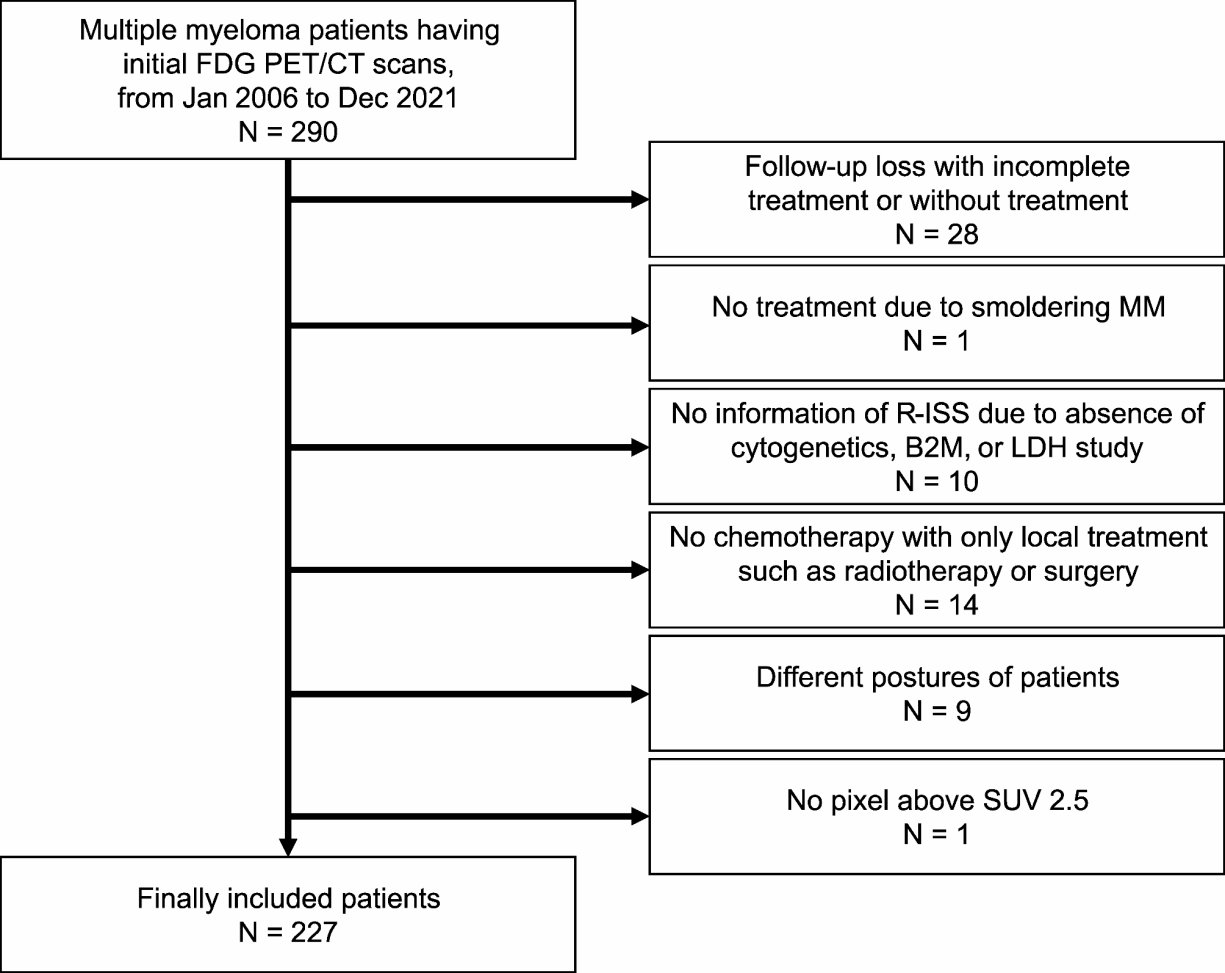
Scheme for selection of study subjects. Two hundred ninety MM patients who underwent FDG PET/CT for initial staging were retrospectively enrolled. We excluded 29 cases who were lost to follow-up without completion of treatment, 14 cases who received local treatment only, and 10 cases for whom R-ISS stage information was unavailable. We additionally excluded nine cases whose PET images did not include the arms and one case that showed low FDG uptake of all bone lesions. Therefore, 227 patients were included in the final analyses. R-ISS: Revised Multiple Myeloma International Staging System

Study candidates were 290 MM patients who underwent FDG PET/CT as an initial workup at our institution between January 2006 and December 2021. Among the candidates, we excluded 28 patients who were lost to follow-up without treatment completion; 14 who received only surgery or local radiotherapy; one patient with smoldering MM; and 10 who did not have cytogenetic, serum β2 microglobulin, or serum LDH results. We additionally excluded nine patients whose PET images did not cover the upper extremities, and one whose bone SUVmax was below 2.5. Thus, 227 patients were included in the final analyses (Fig. 1). All methods were carried out in accordance with relevant guidelines and regulations. Our institutional review board approved this retrospective study (IRB #2022-10-106) and the requirement for written informed consent was waived.

All clinical information and laboratory results were acquired from electronic medical records. Clinical information collected included sex, age, date of diagnosis, chemotherapeutic regimen, ASCT history, date of relapse or progression, and date of death or last follow-up. Laboratory results collected included serum albumin, β2 microglobulin, LDH, calcium, creatinine, hemoglobin level, and cytogenetic study findings. R-ISS stage of each patient was determined based on laboratory test results.

### FDG PET/CT acquisition

All patients fasted for at least six hours and blood glucose level was < 200 mg/dL at the time of PET/CT. Whole-body PET and CT images were acquired 60 min after injection of 5.0 MBq/kg FDG without intravenous or oral contrast on a Discovery LS, a Discovery STE, or a Discovery MI DR PET/CT scanner (GE Healthcare, Milwaukee, WI). Continuous spiral CT was performed with an eight-slice helical CT (140 keV, 40–120 mA, Discovery LS) or 16-slice helical CT (140 keV, 30–170 mA, Discovery STE; 120 keV, 30–100 mA, Discovery MI DR). An emission scan was performed from head to thigh for 4 min per frame in two-dimensional mode (Discovery LS), 2.5 min per frame in three-dimensional mode (Discovery STE), or 2 min per frame in three-dimensional mode (Discovery MI DR). PET images were reconstructed using CT for attenuation correction using the ordered-subsets expectation maximization algorithm with 28 subsets and 2 iterations (matrix 128 × 128, voxel size 4.3 × 4.3 × 3.9 mm, Discovery LS), ordered-subsets expectation maximization algorithm with 20 subsets and two iterations (matrix 128 × 128, voxel size 3.9 × 3.9 × 3.3 mm, Discovery STE), or ordered-subsets expectation maximization algorithm with 18 subsets and four iterations (matrix 192 × 192, voxel size 3.9 × 3.9 × 3.3 mm, Discovery MI DR). The SUV was calculated by adjusting for administered FDG dose and patient body weight.

### FDG PET/CT image analysis

FDG PET/CT images were imported into MIM Encore version 7.0 (MIM Software Inc., Cleveland, OH). For each patient, volumes of interest (VOIs) with Hounsfield units (HU) above 150 were drawn on the CT images from the skull base to the upper thigh. An experienced nuclear medicine physician then carefully reviewed the VOIs and made manual corrections to include the entire skeleton and add osteolytic lesions with low HU while excluding any non-skeletal high-attenuation structures such as calcifications or foreign materials. EMD lesions were not included in the VOIs. The final VOIs and PET images were saved in DICOM format from which SUVmax and mean SUV (SUVmean) were obtained.

A fixed SUV of 2.5 was selected as the threshold for volumetric metabolic parameters of MTV and total lesion glycolysis (TLG) measurements based on a previous study [10]. All voxels with SUVmax greater than 2.5 were included to obtain single MTV and TLG values for each patient, rather than obtaining parameters for individual lesions (nidus). Since malignant cells can be present without structural bone changes, any voxel with increased FDG uptake was included regardless of CT abnormality. Fractures that occurred without a corresponding history of trauma were regarded as pathologic fractures due to MM.

In addition, an experienced nuclear medicine physician additionally recorded the number of FLs, defined as focal FDG uptake higher than that of the liver or normal bone marrow (FL numbers that exceeded 10 were recorded as > 10). Based on a previous study, a FL number of three or greater was considered high [9].

### Survival analysis and statistical methods

The endpoint in this study was overall survival (OS), defined as the duration from date of initial diagnosis to date of any-cause death or last follow-up. Median follow-up was 1,329 days (range, 78 − 6,015 days). An observation was considered right censored and analyzed accordingly if the subject did not have an event at the end of follow-up or was lost to follow-up.

Kaplan-Meier analysis with log-rank tests and univariate Cox proportional hazards models with hazard ratios (HRs) were used to evaluate the prognostic value of variables. Clinical variables were sex, age, R-ISS stage, chemotherapy regimen, radiotherapy, ASCT, hypercalcemia, renal insufficiency, anemia, and the presence of bone lesions or EMD. Age was divided into three ranges, with cut-off points at 57 and 66 years for the entire population. Multivariate Cox analysis was performed using variables with *P* values < 0.05 on univariate analysis. Given its independent prognostic value, subgroup survival analysis was performed according to ASCT.

To determine cut-off values of FDG parameters, those that best discriminated OS were determined by the *surv_cutpoint* function in the *survminer* package of R software. In the non-ASCT cohort, optimum cut-offs for SUVmax, SUVmean, MTV, and TLG were 7.22 g/ml, 3.2 g/ml, 94.2 cm^3^, and 308.04, respectively. Age was divided into three ranges with cut-off points of 67 and 70 years. In the ASCT cohort, respective optimum cut-offs were 6.02 g/ml, 3.04 g/ml, 52.57 cm^3^, and 112.96, respectively. Age was divided into three ranges by cut-off points of 53 and 60 years.

Kaplan-Meier analyses with log-rank tests and univariate Cox proportional regression analyses were performed in the ASCT and non-ASCT cohorts using clinical variables and FDG parameters. In the non-ASCT cohort, FDG parameters showed significant prognostic power, whereas R-ISS stage did not. Therefore, in this cohort, a new three-stage risk system was developed. First, patients in each R-ISS stage were divided into two subgroups based on the values of SUVmax, SUVmean, MTV, TLG, or FL. HRs and 95% confidence intervals were estimated, and log-rank statistics were obtained by the Kaplan-Meier method. Using HRs, the initial six subgroups were arranged into three final risk groups for Kaplan-Meier analysis.

All statistical analyses were performed using R software (v. 4.0.4, https://www.R-project.org/, R Foundation for Statistical Computing, Vienna, Austria) [11]. Two-sided *P* values < 0.05 were considered statistically significant.

## Results

### Demographic data

Among our 227 study subjects, 123 underwent ASCT (54.2%) while 104 did not (45.8%). Clinical characteristics and demographics of the ASCT and non-ASCT cohorts are described in Table 1. The non-ASCT cohort had a median age of 69 years (range: 45–90), 58.7% were male, and 50.0% had R-ISS stage II disease. VMP (bortezomib, melphalan, prednisone) was the most common chemotherapeutic regimen (43.3%), and radiotherapy was performed in 8.7%. Bone lesions were present in 82.7% and anemia in 49.0%. Median SUVmax, SUVmean, MTV, and TLG were 5.73 g/ml, 2.99 g/ml, 31.82 cm^3^, and 115.89, respectively.

**Table 1:** Demographic and clinical characteristics for the entire study subjects according to ASCT.

Characteristics	Patients, n (%)	
	No ASCT (n = 104)	ASCT (n = 123)
Male	61 (58.7)	66 (53.7)
Age*, *range (median)*	*45–90 (69)*	*33–74 (57)*
- Younger	32 (30.8)	40 (32.5)
- Intermediate	37 (35.6)	42 (34.1)
- Older	35 (33.7)	41 (33.3)
R-ISS stage		
- I	33 (31.7)	51 (41.5)
- II	52 (50.0)	53 (43.1)
- III	19 (18.3)	19 (15.4)
Chemotherapy regimen		
- VTD	21 (20.2)	70 (56.9)
- VMP	45 (43.3)	3 (2.4)
- Other three-drug regimens**	5 (4.8)	26 (21.1)
- Miscellaneous	33 (31.7)	24 (19.5)
Radiotherapy	9 (8.7)	18 (14.6)
Hypercalcemia	8 (7.7)	20 (16.3)
Renal insufficiency	24 (23.1)	28 (22.8)
Anemia	51 (49.0)	64 (52.0)
Bone lesion positive	86 (82.7)	110 (89.4)
Extramedullary disease	21 (20.2)	16 (13.0)
FDG parameter***		
- SUVmax, *range (median)*	*2.5–22.3 (5.7)*	*2.5–35.7 (5.5)*
- SUVmean, *range (median)*	*2.6–6.3 (3.0)*	*2.5–7.6 (2.9)*
- MTV, *range (median)*	*0.10–1255.3 (31.8)*	*0.1–1859.2 (33.1)*
- TLG, *range (median)*	*0.26–5058.9 (115.9)*	*0.1–6397.6 (108.8)*
- SUVmax, high	38 (36.5)	80 (65.0)
- SUVmean, high	37 (35.6)	52 (42.3)
- MTV, high	62 (59.6)	48 (39.0)
- TLG, high	34 (32.7)	44 (35.8)
- FL > 3	54 (51.9)	70 (56.9)

ASCT: autologous stem cell transplantation; R-ISS: Revised Multiple Myeloma International Staging System; VTD: bortezomib, thalidomide, dexamethasone; VMP: bortezomib, melphalan, prednisone; SUVmax: maximal standardized uptake value; SUVmean: mean standardized uptake value; MTV: metabolic tumor volume; TLG: total lesion glycolysis; FL: focal lesion. *Cut-offs for age are 67 & 70 years for the non-ASCT cohort and 53 & 60 years for the ASCT cohort, respectively. **Three-drug regimens other than VTD or VMP. ***Cut-offs for high SUVmax, SUVmean, MTV, and TLG are 7.2, 3.2, 94.2. and 308.0 for the non-ASCT cohort and 6.0, 3.0, 52.3, and 113.0 for the ASCT cohort, respectively

There were two different clinical characteristics between the two groups. Firstly, the ASCT cohort was significantly younger (55.5 ± 7.7 vs. 67.9 ± 8.0, *P* < 0.01), which is expected since younger age is generally considered a criterion for ASCT eligibility. Secondly, the ASCT group was more often treated with VTD whereas the non-ASCT group was more often treated with VMP (*P* < 0.01).

### Survival analysis data

In the entire population, univariate survival analysis demonstrated significant associations of age (continuous and categorical; *P* = 0.01 and *P* < 0.05, respectively), chemotherapy regimen (*P* < 0.05), ASCT (*P* < 0.001), and R-ISS stage (overall, *P* < 0.001) with OS (Table 2). Multivariate analysis including these variables revealed ASCT (*P* < 0.001) and R-ISS stage (I vs. II, *P* < 0.05; I vs. III, *P* < 0.001) as independent predictors of OS.

**Table 2:** Cox regression analysis for OS in the entire study population (n = 227)

Variable	Categories	Univariate			Multivariate	
		HR (95% CI)	*P*	Log-rank	HR (95% CI)	*P*
Sex	Male	1.12 (0.71–1.79)	0.629	0.629		
Age	57	1.00		0.010		
	57–66	1.19 (0.63–2.25)	0.586		1.04 (0.52–2.05)	0.920
	≥ 66	2.20 (1.23–3.94)	0.008		0.69 (0.30–1.57)	0.372
*Age (continuous)*	*Per 1 year*	*1.04 (1.01–1.06)*	*0.004*			
Chemotherapy regimen	VTD	1.00		0.017		
	VMP	2.69 (1.43–5.06)	0.002		0.96 (0.42–2.17)	0.917
	Other 3-drugs*	1.93 (0.89–4.16)	0.094		2.22 (0.98–5.01)	0.056
	Miscellaneous	2.03 (1.07–3.86)	0.031		1.33 (0.62–2.85)	0.457
Radiotherapy	Yes	1.11 (0.57–2.17)	0.752	0.751		
ASCT	Yes	0.34 (0.22–0.56)	< 0.001	< 0.001	0.23 (0.11–0.50)	< 0.001
R-ISS stage	I	1.00		< 0.001		
	II	2.34 (1.29–4.25)	0.005		2.06 (1.11–3.79)	0.021
	III	4.19 (2.12–8.26)	< 0.001		4.01 (1.98–8.13)	< 0.001
Hypercalcemia	Yes	0.59 (0.25–1.35)	0.210	0.204		
Renal insufficiency	Yes	1.13 (0.66–1.90)	0.682	0.682		
Anemia	Yes	1.38 (0.87–2.19)	0.173	0.171		
Bone lesions positive	Yes	1.14 (0.57–2.29)	0.717	0.715		
Extramedullary disease	Yes	1.52 (0.87–2.68)	0.152	0.149		

OS: overall survival; HR: hazard ratio; CI: confidence interval; VTD: bortezomib, thalidomide, dexamethasone; VMP: bortezomib, melphalan, prednisone; ASCT: autologous stem cell transplantation; R-ISS: Revised Multiple Myeloma International Staging System. *Three-drug regimens other than VTD or VMP.

**Fig. 2:**
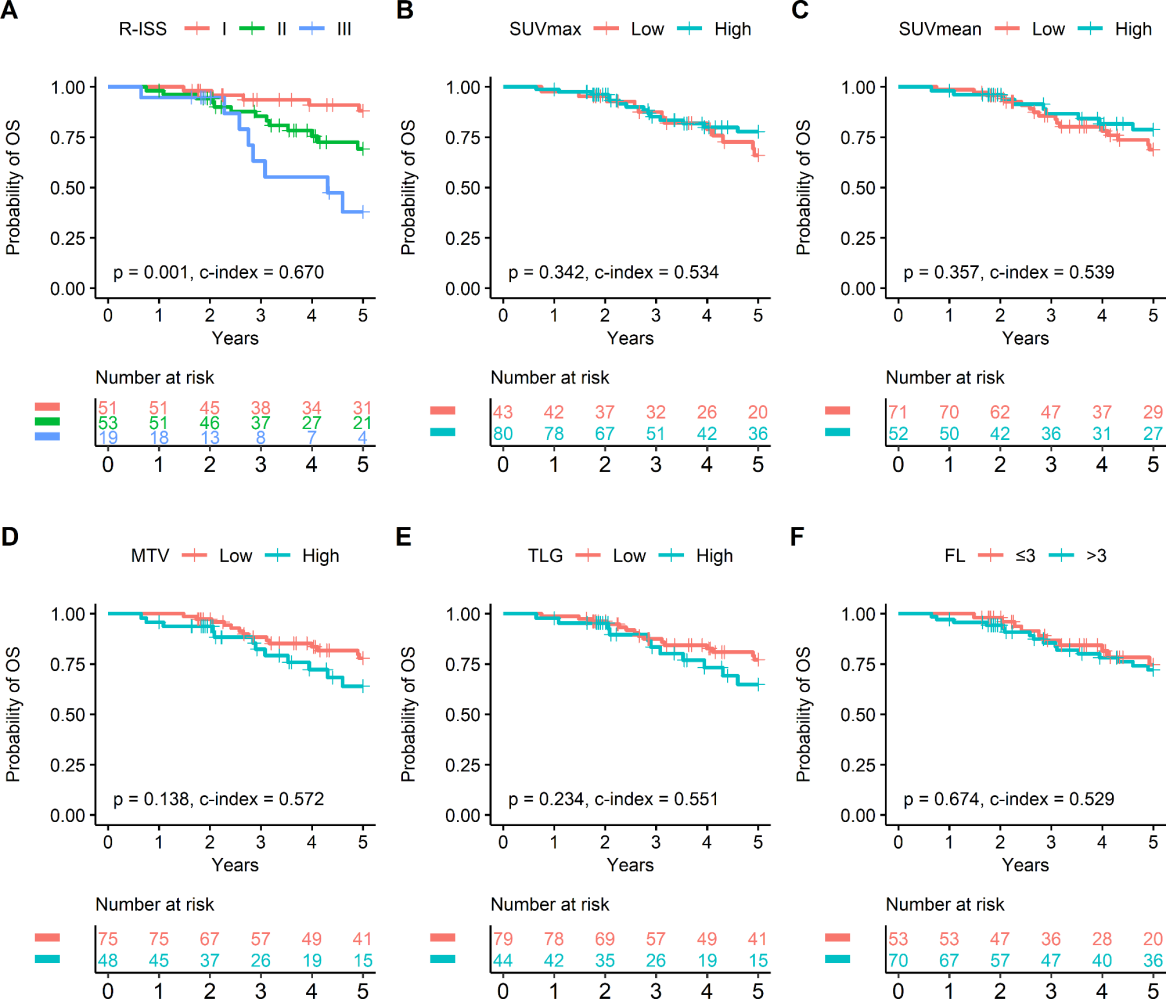
Survival curves in the ASCT cohort. Survival curves based on R-ISS stage effectively stratified patient OS **(A)**. In comparison, SUVmax **(B)**, SUVmean **(C)**, MTV **(D)**, TLG **(E)**, and FL **(F)** failed to adequately stratify prognosis. ASCT: autologous stem cell transplantation; R-ISS: Revised Multiple Myeloma International Staging System; SUVmax: maximal standardized uptake value; SUVmean: mean standardized uptake value; MTV: metabolic tumor volume; TLG: total lesion glycolysis; FL: focal lesion

In the ASCT cohort, R-ISS stage was a strong predictor of OS (*P* = 0.001; Table 3; Fig. 2A). Other significant univariate predictors were chemotherapy regimen (*P* < 0.05), continuous scale MTV (*P* < 0.01) and TLG (*P* < 0.05) (Table 3; Fig. 2B F). However, multivariate analysis showed that VMP regimen (compared to VTD; *P* < 0.05) and R-ISS stage III disease (compared to stage I; *P* < 0.005) were independent predictors of OS in this group (Supplementary Table 1).

**Table 3:** Univariate Cox regression analysis for OS in subjects who received ASCT (n = 123)

Variable	Categories	HR (95% CI)	*P* value	Log-rank
Sex	Male	1.31 (0.60–2.84)	0.503	0.501
Age	< 53	1.00		0.464
	53–60	1.61 (0.63–4.09)	0.317	
	≥ 60	0.99 (0.35–2.81)	0.978	
*Age, continuous*	*Per 1 year*	*1.02 (0.97–1.08)*	*0.397*	
Chemotherapy regimen	VTD	1.00		0.048
	VMP	5.79 (1.23–27.29)	0.026	
	Other 3-drugs*	2.74 (1.06–7.12)	0.038	
	Miscellaneous	2.10 (0.76–5.79)	0.154	
Radiotherapy	Yes	1.66 (0.67–4.14)	0.277	0.272
R-ISS stage	I	1.00		0.001
	II	2.92 (1.04–8.22)	0.042	
	III	6.86 (2.23–21.10)	< 0.001	
Hypercalcemia	Yes	0.64 (0.19–2.19)	0.460	0.456
Renal insufficiency	Yes	1.19 (0.48–2.96)	0.715	0.715
Anemia	Yes	1.36 (0.65–2.97)	0.437	0.435
Bone lesions positive	Yes	0.60 (0.21–1.75)	0.350	0.345
Extramedullary disease	Yes	0.80 (0.24–2.66)	0.712	0.711
**FDG parameter**				
- SUVmax	High	0.69 (0.32–1.49)	0.342	0.339
*- SUVmax, continuous*	*Per 1 g/ml*	*1.06 (0.99–1.13)*	*0.110*	
- SUVmean	High	0.68 (0.31–1.54)	0.357	0.354
*- SUVmean, continuous*	*Per 1 g/ml*	*0.69 (0.35–1.36)*	*0.289*	
- MTV	High	1.80 (0.83–3.90)	0.138	0.132
*- MTV, continuous*	*Per 1 cm* ^*3*^	*1.00 (1.00–1.00)*	*0.009*	
- TLG	High	1.61 (0.74–3.51)	0.234	0.229
*- TLG, continuous*	*Per 1*	*1.00 (1.00–1.00)*	*0.021*	
- FL	> 3	1.19 (0.54–2.62)	0.674	0.674

OS: overall survival; ASCT: autologous stem cell transplantation; HR: hazard ratio; CI: confidence interval; VTD: bortezomib, thalidomide, dexamethasone; VMP: bortezomib, melphalan, prednisone; R-ISS: Revised Multiple Myeloma International Staging System; SUVmax: maximal standardized uptake value; SUVmean: mean standardized uptake value; MTV: metabolic tumor volume; TLG: total lesion glycolysis; FL: focal lesion. *Three-drug regimens other than VTD or VMP.

**Fig. 3:**
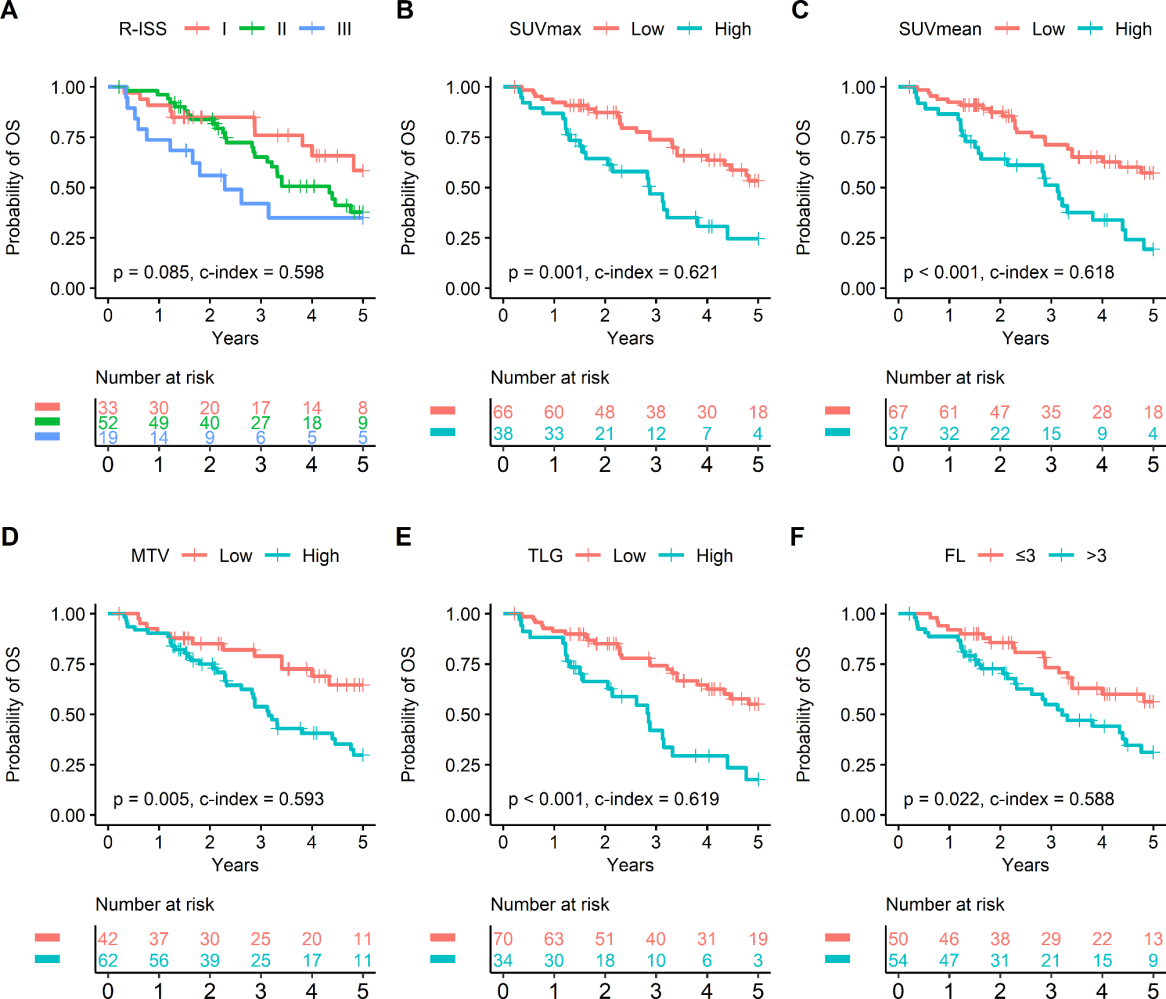
Survival curves in the non-ASCT cohort. For the survival curves according to R-ISS stage, OS between stage I and stage II was not adequately separated, especially during early follow-up. **(A)**. In comparison, SUVmax **(B)**, SUVmean **(C)**, MTV **(D)**, TLG **(E)**, and FL **(F)** effectively stratified patient survival. ASCT: autologous stem cell transplantation; R-ISS: Revised Multiple Myeloma International Staging System; SUVmax: maximal standardized uptake value; SUVmean: mean standardized uptake value; MTV: metabolic tumor volume; TLG: total lesion glycolysis; FL: focal lesion

In contrast, analysis of the non-ASCT cohort showed that none of the clinical variables were significant univariate predictors of OS (Table 4). Although higher R-ISS exhibited a trend for worse survival, the difference did not reach statistical significance (*P* = 0.085). R-ISS proved especially ineffective in stratifying prognosis within the first two-year follow-up (Fig. 3A). In this cohort, however, all FDG parameters including SUVmax (*P* = 0.001), SUVmean (*P* = 0.001), MTV (*P* < 0.005), TLG (*P* < 0.001), and number of FL (*P* < 0.05) were significant prognostic factors (Table 4; Fig. 3B F). Multivariate analysis with significant univariate FDG parameters was not performed because evident multicollinearity between SUV, MTV, and TLG made their simultaneous inclusion inappropriate.

**Table 4:** Univariate Cox regression analysis for OS in subjects who did not receive ASCT (n = 104)

Variable	Categories	HR (95% CI)	*P* value	Log-rank
Sex	Male	0.91 (0.51–1.62)	0.748	0.747
Age	< 67	1.00		0.224
	67–70	0.62 (0.30–1.28)	0.192	
	≥ 70	1.08 (0.54–2.18)	0.821	
*Age, continuous*	*Per 1 year*	*0.99 (0.95–1.02)*	*0.436*	
Chemotherapy regimen	VTD	1.00		0.204
	VMP	0.77 (0.34–1.75)	0.002	
	Other 3-drugs*	3.45 (0.71–16.83)	0.126	
	Miscellaneous	0.82 (0.35–1.94)	0.649	
Radiotherapy	Yes	1.01 (0.36–2.83)	0.979	0.977
R-ISS stage	I	1.00		0.085
	II	1.67 (0.80–3.46)	0.170	
	III	2.59 (1.10–6.11)	0.030	
Hypercalcemia	Yes	0.85 (0.26–2.73)	0.780	0.779
Renal insufficiency	Yes	0.99 (0.51–1.91)	0.973	0.971
Anemia	Yes	1.51 (0.85–2.69)	0.163	0.159
Bone lesions positive	Yes	2.08 (0.82–5.27)	0.122	0.112
Extramedullary disease	Yes	1.81 (0.94–3.50)	0.077	0.073
**FDG parameter**				
- SUVmax	High	2.57 (1.44–4.59)	0.001	0.001
*- SUVmax, continuous*	*Per 1 g/ml*	*1.08 (1.03–1.14)*	*0.002*	
- SUVmean	High	2.65 (1.49–4.72)	0.001	0.001
*- SUVmean, continuous*	*Per 1 g/ml*	*1.77 (1.25–2.49)*	*0.001*	
- MTV	High	2.51 (1.30–4.84)	0.006	0.005
*- MTV, continuous*	*Per 1 cm* ^*3*^	*1.00 (1.00–1.00)*	*0.002*	
- TLG	High	2.84 (1.59–5.08)	< 0.001	< 0.001
*- TLG, continuous*	*Per 1*	*1.00 (1.00–1.00)*	*0.001*	
- FL	> 3	1.99 (1.10–3.59)	0.022	0.020

OS: overall survival; ASCT: autologous stem cell transplantation; HR: hazard ratio; CI: confidence interval; VTD: bortezomib, thalidomide, dexamethasone; VMP: bortezomib, melphalan, prednisone; R-ISS: Revised Multiple Myeloma International Staging System; SUVmax: maximal standardized uptake value; SUVmean: mean standardized uptake value; MTV: metabolic tumor volume; TLG: total lesion glycolysis; FL: focal lesion. *Three-drug regimens other than VTD or VMP.

### Prognostic significance of the new staging system that includes FDG parameters

Given the limited prognostic value of R-ISS stage in the non-ASCT cohort and the significant prognostic associations of FDG parameters in this group, we explored how combining the two types of variables might improve risk stratification. For SUVmax, the results showed that the HR of ‘R-ISS II/low SUVmax’ subgroup was not different from that of the ‘R-ISS I/low SUVmax’ subgroup (*P* = 0.244), whereas it was greater for ‘R-ISS II/high SUVmax’ and ‘R-ISS III/high SUVmax’ subgroups (*P* = 0.010 and < 0.001; respectively; Supplementary Table 2). Similarly, HR did not differ between ‘R-ISS I/high SUVmax’ and ‘R-ISS II/high SUVmax (*P* = 0.621) or ‘R-ISS III/low SUVmax’ subgroups (*P* = 0.749; data not shown). These results led to the proposal of a new risk classification system in which R-ISS II/low SUVmax subjects are down-staged into new stage I; and R-ISS I/high SUVmax and R-ISS III/low SUVmax subjects are up-staged and down-staged, respectively, into new stage II. Similar reclassification of risk was possible by combining R-ISS stage with SUVmean, MTV, TLG, or FL (Supplementary Table 2).

**Fig. 4:**
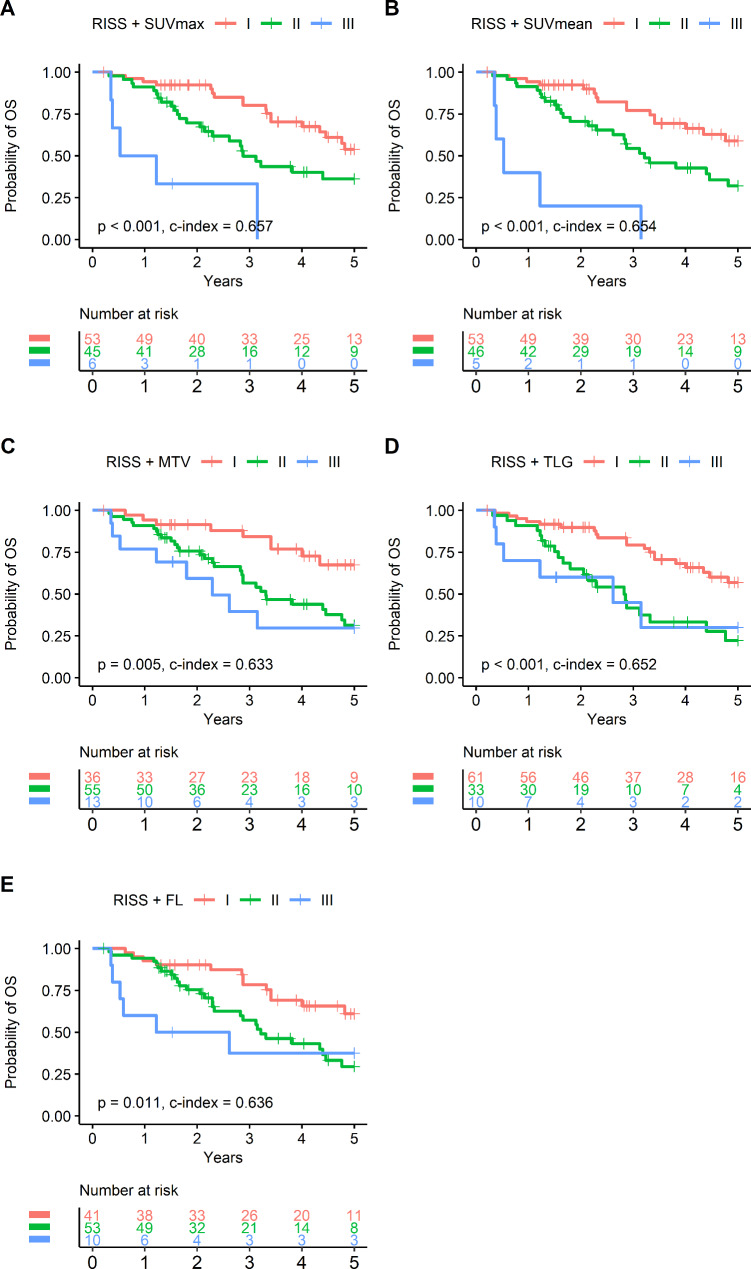
Survival curves in the non-ASCT cohort according to the newly developed risk staging system. Compared with survival curves based on conventional R-ISS staging, the new proposed system combined FDG parameters with R-ISS staging and provided better prognostic discrimination. Staging that incorporated SUVmax **(A)** or SUVmean **(B)** effectively differentiated survival outcomes across stages. Staging systems that incorporated MTV **(C)**, TLG **(D)**, or FL **(E)** also yielded significantly improved risk stratification

Cox regression and survival curve analysis based on the new risk classification system confirmed significantly improved stratification of OS compared to that based on R-ISS stage alone (Table 5; Fig. 4). Indeed, combining any of the FDG parameters provided new risk stage I and II groups with significantly different survival outcomes. Furthermore, combining SUVmax or SUVmean allowed effective stratification of outcomes between new stage II and III groups (*P* = 0.007 and < 0.001; respectively; Table 5).

**Table 5:** Univariate Cox regression for OS in the non-ASCT cohort according to the new risk stage

FDG parameter	New risk stage	HR (95% CI)	*P* value	Log-rank
R-ISS + SUVmax	Overall			< 0.001
	I vs. II	2.148 (1.161–3.974)	0.015	
	I vs. III	9.617 (3.438–26.902)	< 0.001	
	II vs. III	3.922 (1.459–10.540)	0.007	
R-ISS + SUVmean	Overall			< 0.001
	I vs. II	2.262 (1.211–4.223)	0.010	
	I vs. III	12.658 (4.487–35.713)	< 0.001	
	II vs. III	5.423 (2.005–14.670)	< 0.001	
R-ISS + MTV	Overall			0.005
	I vs. II	2.806 (1.329–5.924)	0.007	
	I vs. III	3.929 (1.510–10.222)	0.005	
	II vs. III	1.399 (0.640–3.056)	0.400	
R-ISS + TLG	Overall			< 0.001
	I vs. II	2.888 (1.555–5.363)	< 0.001	
	I vs. III	3.178 (1.272–7.941)	0.013	
	II vs. III	1.125 (0.453–2.796)	0.799	
R-ISS + FL	Overall			0.014
	I vs. II	2.306 (1.190–4.468)	0.013	
	I vs. III	3.168 (1.201–8.358)	0.020	
	II vs. III	1.379 (0.568–3.349)	0.478	

OS: overall survival; ASCT: autologous stem cell transplantation; HR: hazard ratio; CI: confidence interval; R-ISS: Revised Multiple Myeloma International Staging System; SUVmax: maximal standardized uptake value; SUVmean: mean standardized uptake value; MTV: metabolic tumor volume; TLG: total lesion glycolysis; FL: focal lesion

## Discussion

This study compared the value of FDG parameters and clinical variables for stratifying risk in MM patients. In patients who did not receive ASCT, R-ISS stage showed limited prognostic value, but SUVmax, SUVmean, MTV, TLG, and FL number demonstrated significant associations with OS. This enabled us to combine FDG parameters with R-ISS stage as a new risk classification system that better discriminated poor OS in these patients compared to R-ISS stage alone.

ASCT is a standard treatment option that improves the outcomes of eligible MM patients [1]. Our finding of better survival for patients who received ASCT is consistent with this notion. In patients who did not receive ASCT, effective risk stratification was not achieved by R-ISS stage alone, even though it is the standard prognostic tool in MM. Possibly related to this finding includes the fact that patients not eligible for ASCT are older and/or have more comorbidities associated with aggressive or treatment-resistant disease. Failure to receive ASCT itself often contributes to further worsening of treatment response. Importantly, these differences may not be fully accounted for by R-ISS staging. This underlines the need for newer prognostic factors such as FDG parameters that can improve risk stratification of MM patients who do not receive ASCT.

FDG PET/CT provides whole-body images of cancer cells with enhanced glucose metabolism, reflecting the burden of aggressive tumor tissue. The prognostic value of FDG PET/CT in various malignancies is well established. In MM, high SUV, MTV, and FL number have shown significant associations with worse prognosis [7–9]. A few previous studies reported that post-ASCT FDG PET/CT findings were correlated with patient outcomes [12, 13]. A recent study on cluster analysis of FDG PET/CT images observed as an ancillary finding that the prognostic value of MTV differed between MM patients with and without ASCT [14]. However, the prognostic value of FDG PET/CT parameters in MM according to ASCT has not been fully explored.

In this study, survival analysis was separately performed for patients with and without ASCT. In the ASCT cohort, sufficient outcome prediction was provided by R-ISS stage with no significant prognostic information offered by any of the FDG parameters. The latter finding is divergent from that observed in a previous study where MM patients who received ASCT demonstrated worse outcomes in the presence of higher SUVmax (> 4.2), FL (> 3), and EMD [7]. In our ASCT cohort, neither FL (> 3) nor EMD was associated with patient survival. Even high SUVmax defined as > 4.2 as in the above paper failed to significantly stratify prognosis in this cohort (data not shown). Given the relatively favorable OS in this group, it is possible that the robust prognostic association with R-ISS leaves little room for additional prognostic information provided by FDG parameters.

In the non-ASCT cohort, by contrast, all semi-quantitative FDG parameters showed significant associations with patient outcomes, while R-ISS stage did not. The survival outcome associations of MTV and TLG are not surprising as these variables reflect tumor volume and overall glycolysis, respectively, and have well-established prognostic value in various malignancies. Interestingly, SUVmax and SUVmean, despite being non-volumetric FDG parameters, also showed significant associations with patient outcome. Furthermore, even the semi-quantitative parameter of FL number demonstrated prognostic value. Multivariate analysis in the non-ASCT cohort was not performed because none of the clinical variables were significant univariate predictors of survival. As for image parameters, although significant prognostic correlations were shown on univariate analysis, evident multicollinearity between SUV, MTV, and TLG made it inappropriate to include them for multivariate analysis.

Based on the above findings, we investigated whether combining FDG parameters with R-ISS stage could improve risk stratification for the non-ASCT cohort. As a result, FDG parameters could subcategorize subjects with significantly different risks among R-ISS stages. In addition, incorporating FDG parameters improved stratification of early risk between new stage I and II groups that had been limited by conventional R-ISS staging.

It is noteworthy that incorporating SUVmax into the new staging system yielded excellent risk stratification that outperformed that provided by MTV or TLG. This finding may indicate that the survival outcome of MM patients is substantially contributed by the most aggressive tumor cells, which likely has the highest FDG uptake. It should also be noted that, the volumes of MTV and TLG in MM patients encompass not only malignant cells but also normal and hyperplastic marrow cells, which might have diminished their prognostic efficacy compared to that of SUVmax.

Although incorporating FL number also helped categorize survival risk, it was less effective for discriminating risk between stage II and III groups. A previous study showed that FL number allowed stage readjustment with improved prognostic capacity in MM [9]. A major difference is that our analysis was in patients without ASCT, who typically have a worse prognosis.

In our results, combining FDG parameters with R-ISS did not significantly improve the ability to predict PFS. Therefore, primary endpoint of this study was focused on OS, which has a more precise criteria and event occurrence date compared to PFS that sometimes has a less-than-clear criteria and occurrence date.

Taken together, our results demonstrate that the prognostic value of FDG parameters in MM differs according to ASCT, with greater value in patients without the treatment. They also reveal that incorporating SUVmax, SUVmean, MTV, TLG, or FL number improves risk classification compared to that based on R-ISS stage alone. The unexpectedly strong association of SUVmax with patient outcomes has particularly important practical implications because this simple FDG parameter is substantially easier to measure than MTV or TLG and is virtually free from inter-observer variability. Thus, the routine measurement of SUVmax in daily clinical practice could help select MM patients without ASCT at higher risk who would benefit from shorter follow-up intervals and more refined treatment.

Limitations of this study include that PET/CT imaging was confined to the torso, although most osseous myeloma lesions are in this region. Another limitation is that only patients with lesions of SUVmax above 2.5 were included for analysis. However, considering that the SUV of aggressive lesions is generally high, we do not think that this would have significantly compromised the conclusions of our study. In addition, the inclusion of three types of PET/CT scanners could potentially influence SUV measurements, and analysis in our study was performed without an integration process. However, a previous phantom study showed that SUV results across varying PET/CT instruments were not significantly different [15].

## Conclusions

In conclusion, we demonstrated that FDG parameters were significant predictors of worse OS in MM patients without ASCT. This led to the development of a new risk classification system that combines FDG parameters with R-ISS stage, which improved prognostic stratification compared to that by R-ISS stage alone. Future prospective investigations are thus warranted to verify whether this new staging system may help treatment decisions and enhance the management of MM patients who have not received ASCT.

### Electronic supplementary material

Below is the link to the electronic supplementary material.

Supplementary Material 1

## Data Availability

The data generated in this study are available upon request from the corresponding author.

## Abbreviations

MM: multiple myeloma
ISS: International Staging System
R-ISS: Revised Multiple Myeloma International Staging System
LDH: lactate dehydrogenase
F-18 FDG PET/CT: F-18 fluorodeoxyglucose positron emission tomography/computed tomography
EMD: extramedullary disease
SUV: standardized uptake value
SUVmax: maximum SUV
MTV: metabolic tumor volume
FL: focal lesion
ASCT: autologous stem cell transplantation
VOI: volume-of-interest
HU: Hounsfield units
SUVmean: mean SUV
TLG: total lesion glycolysis
OS: overall survival
HR: hazard ratio
