# Supplementary material for: Semi-quantitative FDG parameters predict survival in multiple myeloma patients without autologous stem cell transplantation
Source: Cancer Imaging. 2023 Oct 27;23:104. doi: 10.1186/s40644-023-00625-z (PMC10612180; doi:10.1186/s40644-023-00625-z)
Supplement: Supplementary file 1 — Supplementary Material 1 [file 40644_2023_625_MOESM1_ESM.docx]

**SUPPLEMENTARY TABLES**

**Supplementary Table 1.** Multivariate Cox regression for OS in the ASCT cohort.

| **Variable** | **Categories** | **MTV model** | | **TLG model** | |
| --- | --- | --- | --- | --- | --- |
|  |  | **HR (95% CI)** | ***P*** | **HR (95% CI)** | ***P*** |
| Chemotherapy regimen | VTD | 1.00 |  | 1.00 |  |
|  | VMP | 5.44 (1.08–27.42) | 0.040 | 5.42 (1.08–27.30) | 0.406 |
|  | Other 3-drugs* | 2.20 (0.83–5.82) | 0.111 | 2.26 (0.86–5.95) | 0.099 |
|  | Miscellaneous | 2.30 (0.82–6.46) | 0.115 | 2.38 (0.85–6.65) | 0.099 |
| R-ISS stage | I | 1.00 |  | 1.00 |  |
|  | II | 2.23 (0.76–6.58) | 0.146 | 2.24 (0.76–6.60) | 0.145 |
|  | III | 5.95 (1.84–19.30) | 0.003 | 6.11 (1.90–19.67) | 0.002 |
| MTV | Per 1 cm^3^ | 1.00 (1.00–1.00) | 0.096 |  |  |
| TLG | Per 1 |  |  | 1.00 (1.00–1.00) | 0.113 |

OS, overall survival; ASCT, autologous stem cell transplantation; MTV, metabolic tumor volume; TLG, total lesion glycolysis; HR, hazard ratio; CI, confidence interval; R-ISS, Revised Multiple Myeloma International Staging System; VTD, bortezomib, thalidomide, dexamethasone; VMP, bortezomib, melphalan, prednisone. *Three-drug regimens other than VTD or VMP.

**Supplementary Table 2.** Univariate Cox regression analysis for OS according to R-ISS/FDG category.

| **R-ISS stage** | **FDG category*** | **SUVmax** | | **SUVmean** | | **MTV** | | **TLG** | | **FL** | |
| --- | --- | --- | --- | --- | --- | --- | --- | --- | --- | --- | --- |
|  |  | **HR** | ***P*** | **HR** | ***P*** | **HR** | ***P*** | **HR** | ***P*** | **HR** | ***P*** |
| I | Low | 1.00 |  |  |  |  |  |  |  |  |  |
|  | High | 2.98 | 0.085 | 3.03 | 0.086 | 3.16 | 0.096 | 4.90 | 0.022 | 1.81 | 0.361 |
| II | Low | 1.85 | 0.244 | 1.93 | 0.254 | 1.75 | 0.431 | 1.52 | 0.371 | 1.21 | 0.729 |
|  | High | 3.93 | 0.010 | 4.57 | 0.008 | 4.28 | 0.019 | 3.96 | 0.004 | 2.71 | 0.034 |
| III | Low | 2.43 | 0.143 | 2.63 | 0.134 | 4.00 | 0.090 | 2.86 | 0.073 | 2.84 | 0.086 |
|  | High | 14.56 | <0.001 | 20.09 | <0.001 | 5.50 | 0.012 | 4.09 | 0.012 | 3.50 | 0.031 |

OS, overall survival; R-ISS, Revised Multiple Myeloma International Staging System; HR, hazard ratio; SUVmax, maximal standardized uptake value; SUVmean, mean standardized uptake value; MTV, metabolic tumor volume; TLG, total lesion glycolysis; FL, focal lesion. *For FL, low indicates ≤ 3, and high indicates > 3. All statistical comparisons are with the R-ISS stage I, FDG low category.
